# Transcriptomics and metabolomics revealed that phosphate improves the cold tolerance of alfalfa

**DOI:** 10.3389/fpls.2023.1100601

**Published:** 2023-03-09

**Authors:** Yuntao Wang, Zhen Sun, Qiqi Wang, Jihong Xie, Linqing Yu

**Affiliations:** ^1^ Grassland Research Institute, Chinese Academy of Agricultural Science, Hohhot, Inner Mongolia, China; ^2^ College of Grassland, Qingdao Agricultural University, Qingdao, Shandong, China; ^3^ College of Life Science, Inner Mongolia University, Hohhot, Inner Mongolia, China

**Keywords:** alfalfa, transcriptomics, metabolomics, cold tolerance, phosphorus

## Abstract

**Introduction:**

Alfalfa (*Medicago sativa L.*) is a highly nutritious leguminous forage that plays an essential role in animal husbandry. In the middle and high latitudes of the northern hemisphere, there are problems with its low rates of overwintering and production. The application of phosphate (P) is an important measure to improve the cold resistance and production of alfalfa, but little is known about the mechanism of P in improving the cold resistance of alfalfa.

**Methods:**

This study integrated the transcriptome and metabolome to explain the mechanism of alfalfa in response to low-temperature stress under two applications of P (50 and 200 mg kg^-1^) and a control of none applied.

**Results:**

The application of P fertilizer improved the root structure and increased the content of soluble sugar and soluble protein in the root crown. In addition, there were 49 differentially expressed genes (DEGs) with 23 upregulated and 24 metabolites with 12 upregulated when 50 mg kg^-1^ of P was applied. In contrast, there were 224 DEGs with 173 upregulated and 12 metabolites with 6 upregulated in the plants treated with 200 mg kg^-1^ of P compared with the Control Check (CK). These genes and metabolites were significantly enriched in the biosynthesis of other secondary metabolites and the metabolic pathways of carbohydrates and amino acids. The integration of the transcriptome and metabolome indicated that P affected the biosynthesis of N-acetyl-L-phenylalanine, L-serine, lactose, and isocitrate during the period of increasing cold. It could also affect the expression of related genes that regulate cold tolerance in alfalfa.

**Discussion:**

Our findings could contribute to a deeper understanding of the mechanism that alfalfa uses to tolerate cold and lay a theoretical foundation for breeding alfalfa that is highly efficient at utilizing phosphorus.

## 1 Introduction

As a cool-season perennial forage crop, alfalfa (*Medicago sativa* L.) plays a variety of beneficial roles for livestock, soil coverage, soil fertility, and human health (Gaafar et al., 2019). It has a high biomass yield, excellent nutritional quality, and wide adaption, and it can fix nitrogen (N) (Summers and Putnam, 2008). Alfalfa is grown on approximately 30 million ha of land worldwide (Acharya et al., 2020; Diatta et al., 2021), and the global production of alfalfa hay was 210.9 million metric tons (Edde, 2022). North America is the primary producer of alfalfa in the world; the United States has the largest planting area with more than 7.23 million ha of alfalfa grown annually, with Canada and Mexico close behind (Putnam et al., 2001; Wechsler et al., 2017; Edde, 2022). The cultivated area of alfalfa in China is approximately 3.77 × 10^6^ hm^2^, which is approximately 13% of the world’s total arable land used to grow alfalfa. The growth of alfalfa is primarily distributed in northeastern, northwestern, and northern China (Yang et al., 2021). In these northern regions, the inability of alfalfa to withstand low subfreezing temperatures remains one of its biggest challenges (Castonguay et al., 2006; Rocher et al., 2015), which hampers the development of the alfalfa industry and animal husbandry.

The morphological and physiological characteristics of the roots are strongly associated with the cold resistance of alfalfa. The root types of alfalfa primarily include four types: tap, branch, rhizomatous, and creeping roots (Goplen, 1987; Hong et al., 1987). Tap-rooted types have a main root and a narrow, protruding crown; the branch-rooted types of alfalfa have a moderately wide crown and a number of primary roots; the rhizomatous- and creeping-rooted types have a protected crown, develop new roots readily, and can also develop stems from the roots. The shoots can separate from the maternal parent plant and become independent plants to survive; thus, they more easily regrow after freezing damage and show strong cold resistance (Goplen, 1987; Dou, 2011). A substantial amount of research has documented that the root crown, lateral roots, and root biomass affect the winter survival and persistence of alfalfa (Castonguay et al., 2006; Liu et al., 2015b; Xu et al., 2022). Previous studies have primarily focused on the root crown. It is a transitional plant structure that is located between the shoots and the root system. It is also the uppermost dormant organ of the winter plant body, which is crucial to overwintering and regeneration in the spring (Marquez-Ortiz et al., 1999; Liu et al., 2015b). The overwintering rate of alfalfa is closely related to the size and depth of the root crown; they grow more deeply and thicker in the soil, which helps the plants to successfully survive the winter (Wang, 2021b). This is considered to be a cold-sensitivity escape mechanism to prevent the exposure of the overwintering organ to low temperature. The major alfalfa varieties with strong cold resistance have more lateral roots because they help alfalfa plants to absorb more water and nutrient elements from the soil to supply the demand of overwintering plants for nutrients (Johnson et al., 1996; Wang et al., 2023). The root biomass is related to the accumulation of organic matter, and the herringbone branching is conducive to the absorption of deeper water by alfalfa, which contributes to improve the plant’s cold resistance (Viands, 1988). Its physiology, including the contents of soluble sugar and protein, is also closely related to cold resistance. Soluble sugar acts as an osmotic regulator, a cryoprotectant, and a signaling molecule to stabilize the cell membrane and scavenge reactive oxygen species (ROS) under low-temperature stress, and its contents increase during the freezing process to protect the plant cells (Trischuk et al., 2014). Therefore, the accumulation of sugar at the root crown before winter is related to the cold resistance of alfalfa (Cunningham et al., 1998; Bertrand et al., 2017). Soluble proteins are strongly hydrophilic and can enhance the water-holding capacity of cells. The accumulation of soluble proteins can bind more water to the cells and reduce the damage caused by low temperatures (Kontunen-Soppela et al., 2002; Moieni-Korbekandi et al., 2013). The increase in the soluble protein content of alfalfa in autumn and winter helps to enhance its resistance to low temperatures.

As an important means of regulating cultivation, nutrient management not only can improve the production of alfalfa, but it is also an essential measure to improve its stress resistance (Wang, 2021a). Phosphorus is one of the three essential nutrients for plant growth and development, which is absorbed in the form of phosphate (P) (Bhosale et al., 2018). It is an essential macroelement that plays a role in an array of processes, including energy generation, nucleic acid synthesis, photosynthesis, glycolysis, respiration, membrane synthesis and stability, respiration, carbohydrate metabolism, and N fixation (Vance et al., 2003; Raghothama and Karthikeyan, 2005). In addition, it plays a vital role in enhancing the adaptability of plants to the external environment (Yan et al., 2022). P is also an important component of phospholipids and ATP, which affect the resistance of plants to low temperatures (Li et al., 2016). Phospholipids in the plant cell membranes can interact with proteins, sugars, and other substances to alleviate the cell dehydration caused by low-temperature stress and protect the stability of cell membranes (Cheng, 2008). Plants can be negatively affected by the low levels of phosphorus that affect a variety of growing environments, particularly in soils that are calcareous or alkaline (Yue et al., 2019). A deficiency in P will affect plant photosynthesis, such as by limiting the distribution and utilization of carbohydrates and the absorption and transport of P (Terry and Tao, 1991). This can also cause an imbalance in the production and clearance of ROS and an array of physiological, biochemical, and metabolic changes resulting in damage to the plants (Lin et al., 2010). The application of P can increase the contents of soluble sugar in plants, and sucrose transport in the phloem requires ATP hydrolysis to provide energy so that P can regulate the metabolism and transport of sucrose in plant leaves (Tian et al., 2017). Current studies have shown that the application of moderate P fertilizer can promote the root growth of alfalfa and increase the content of cold-resistant substances, such as starch and soluble proteins, in the root crown of alfalfa to some extent (Shen et al., 2017). However, the related molecular mechanism remains unclear.

The recently developed technologies of high-throughput sequencing, high-resolution mass spectrometry, and information processing have made systems biology (omics) research a major focus of scientific investigation (Xin et al., 2019). The integrated analyses of transcriptomic and metabolic data obtained from two biological levels, i.e., transcript and metabolite levels, respectively, can serve as a useful way to study complex biological phenomena (Agarrwal et al., 2016). In addition, these integrated analyses have been applied to various plant biological processes, including the evolutionary adaption of poplar species (*Populus* L.) to salinity stress (Janz et al., 2010), rice (*Oryza sativa* L.) insect interaction research (Agarrwal et al., 2016), and the response of wild soybean (*Glycine max* L.) to N starvation (Liu et al., 2020). However, to our knowledge, there are few studies that have examined the mechanisms of the effect of P fertilizer on the cold resistance of alfalfa by integrating the analyses of transcriptomic and metabolic data.

In this study, alfalfa was exposed to three P treatments for 90 days. We measured the contents of the soluble sugar and protein of root crowns and some root indices. In addition, we performed transcriptomic and metabolomic studies among the three P treatments under cold temperature. Our integrated transcriptome–metabolome analysis allowed us to gain a deeper understanding of alfalfa’s response to the application of P in terms of cold tolerance. We sought to identify the strategies used by alfalfa to respond to the application of P during the increase in cold stress, which could be used to improve the cold tolerance and yield of alfalfa and breed new alfalfa varieties.

## 2 Materials and methods

### 2.1 Field design and sampling

The variety of alfalfa used in this study was Zhongcao No. 3, which was provided by the Grassland Research Institute of the Chinese Academy of Agricultural Sciences (GRI of CAAS) (Beijing, China). On September 5, 2020, an alfalfa plant that had grown for 5 years in the Agro-pastoral Experiment Station (40°34′E; 111°45′N; 1,050 m a.s.l.) of the GRI of CAAS was transferred to the greenhouse, and the seedlings were raised by stem cuttings in seedling trays on May 10, 2021. On July 15, 2021, cloned plants were selected and transplanted into pots that were 18 cm in diameter and 19 cm in depth. Each pot had only one plant and 5 kg of gray cinnamon soil from the field as a substrate. The physiochemical properties of the soil included an organic matter content of 6.25 g kg^-1^, total N content of 1.09 g kg^-1^, available N content of 69.45 mg g^-1^, available P content of 20.5 mg kg^-1^, available potassium content of 425.0 mg kg^-1^, and a pH of 8.5. Three levels of P levels were established according to P_2_O_5_ contents of 0, 50, and 200 mg kg^-1^ (designated CK, P1, and P4, respectively). Each treatment had 10 pots, which were placed into the field. The root crowns of alfalfa were collected on October 15, 2021. Three clones served as biological replicates. The root crowns were rinsed with clean water, wiped with absorbent paper, cut into small pieces of 3–5 mm, quickly placed into frozen tubes, stored in liquid N, and then preserved in at -80°C for further analysis (Ye et al., 2018). The temperature during the sampling period is shown in **Figure S1**.

### 2.2 Analyses of morphological and physiological indices

The phenotypic characteristics of alfalfa were measured as previously described (Wang et al., 2022). The root dry weight was measured by weighing the dried roots, which were oven-dried at 105°C for 30 min and then at 65°C for 48 h. The contents of soluble sugar were determined using anthrone–sulfuric acid colorimetry (Yemm and Willis, 1954). The contents of soluble protein were determined using Coomassie brilliant blue (Wu et al., 2013). Three plants were used as biological replicates for each treatment.

### 2.3 RNA extraction and sequencing

Samples treated with different levels of P were fully ground in liquid N. Total RNA was extracted using the TRIzol reagent (Life Technologies, Carlsbad, CA, USA) according to the manufacturer’s instructions. The RNA was purified, reverse-transcribed, used to construct a library, and sequenced according to the manufacturer’s instructions. To prepare Illumina RNA-seq libraries, the poly(A) mRNA was isolated from purified total RNA using biotin-Oligo (dT) magnetic beads and fragmented into small pieces using an RNA fragmentation kit according to the manufacturer’s instructions (Illumina, San Diego, CA, USA) (Hu et al., 2020). Fragmented RNA was then used as a template, and random oligonucleotides were used as primers to synthesize single-stranded complementary DNA (cDNA) by reverse transcription (Du, 2021). RNase H was then used to degrade the RNA strands, and a QiaQuick PCR extraction kit (Qiagen, Venlo, The Netherlands) was used to synthesize double-stranded cDNA (Du, 2021). The purified double-stranded cDNA was repaired by terminal repair. A tail was added and connected to the sequencing junction, and the cDNA that was approximately 200 bp was screened by ampure (AM) Pure Extraction-Purification (XP) beads. PCR amplification was conducted, and the PCR product was purified by AM Pure XP beads, which provided the cDNA library used for sequencing (Bao et al., 2020). To ensure the sequencing quality, a library quality inspection kit, the DNA 1000 assay kit (Agilent Technologies, Santa Clara, CA, USA), was used to inspect the library. The amplified fragments were sequenced using an Illumina Hi-Seq 4000 platform.

The reads obtained from the sequencing machines included raw reads that contained adapters or low-quality bases that would affect the following assembly and analysis. Thus, the reads were further filtered using an ultra-fast all-in-one FASTQ preprocessor (FASTP) (version 0.18.0) to obtain high-quality clean reads (Chen et al., 2018). An index of the reference genome was built, and paired-end clean reads were mapped to the reference genome using HISAT2. 2.4 with “-RNA-strandness RF” and other parameters set as the default (Kim et al., 2015; Chen et al., 2020). By comparing the value of the transcript on each Unigene, the value was standardized to the fragments per kilobase of transcript per million fragments mapped (FPKM) to represent the level of gene expression (Zhao et al., 2021). RNA differential expression analysis was performed using DESeq2 and edgeR (Robinson et al., 2010; Love et al., 2014). Genes with a false discovery rate below 0.05 and an absolute fold change ≥2 were considered to be differentially expressed genes (DEGs). Based on the expression information, we used R^1^ to conduct principal component analysis (PCA) and hierarchical clustering analyses. Gene Ontology (GO) functional and Kyoto Encyclopedia of Genes and Genomes (KEGG) pathway enrichment analyses were performed using GOATOOLS and KoBAS software, which can determine the primary biological functions of DEGs and identify the main biological pathways in which the DEGs are involved (Zhang et al., 2021). All the RNA sequencing data reported in this study have been deposited in National Center for Biotechnology Information (NCBI) under the sequence read archive with the accession number PRJNA909902.

The same batch of samples used in transcriptome sequencing was used for quantitative PCR (qPCR) detection. The qPCR primers and genes are shown in **Table S1**. The *AKR4C9* gene was used as a reference. Real-time quantitative reverse transcription PCR (qRT-PCR) was conducted using a qTOWER 2.2 PCR System (Jena, Germany) and SYBR Green PCR Master Mix (TaKaRa, Shiga, Japan). The amplification program was as follows: 90 s at 95°C, and 40 cycles × (95°C, 5 s, 60°C, 15 s, and 72°C, 20 s). The dissolution curve of the amplified product was analyzed at 65°C–95°C. Each reaction was performed three times. The levels of the expression of candidate genes were measured using the 2^-(ΔΔCt)^ method.

### 2.4 Metabolite extraction and ultra-high-performance liquid chromatography–quadrupole time-of-flight mass spectrometry

The samples treated with different levels of P were removed from storage at -80°C, vacuum frozen-freeze dried, and then crushed using a mixer mill (MM 400, RETSCH, Haan, Germany) with a zirconia bead for 1.5 min at 30 Hz. The sample (100 mg powder) was extracted overnight at 4°C with 1.0 ml of 70% aqueous methanol that contained 0.1 mg/L lidocaine as an internal standard. The supernatant was absorbed after centrifugation at 10,000 g for 10 min (CNWBOND Carbon-GCB SPE Cartridge, 250 mg, 3 ml; ANPEL, Shanghai, China, www.anpel.com.cn/cnw) and filtered (SCAA-104, 0.22 μm pore size; ANPEL) before liquid chromatography with tandem mass spectrometry (LC-MS/Metabolites (MS)) analysis (Chen et al., 2013). After the metabolites were extracted, 10 µl of each sample were mixed to serve as quality control (QC) samples, and 60 µl were evaluated by ultra-high-performance liquid chromatography–quadrupole time-of-flight mass spectrometry (UPLC-QTOF-MS) to monitor the stability of the instrument during the whole analytical process (Zhang et al., 2021).

The metabolites of the samples were qualitatively analyzed by mass spectrometry based on the metabolite database assembled by Guangzhou Chideo Biotechnology Co., Ltd. (Guangzhou, China). The structural analysis of some metabolites refers to the secondary Metabolites (MS) information in the existing mass spectrometry public databases, such as MassBank^2^, HMDB^3^ (Wishart et al., 2013), MoToDB^4^, and METLIN^5^ (Zheng et al., 2013). The quantification of metabolites was conducted by integrating the peak area of the mass spectra of all the substances and correcting the mass spectrometry peaks of the same metabolite in different samples to ensure the accuracy of quantification. The metabolites were analyzed by a PCA, partial least squares discriminant analysis, and orthogonal partial least squares discriminant analysis. The differences in metabolites between the different treatments were analyzed by a *t*-test and one-way analysis of variance (ANOVA; Zhao et al., 2020). The metabolites were mapped to the KEGG metabolic pathways for pathway and enrichment analyses.

### 2.5 Statistical analysis

The data were analyzed statistically with Statistical analysis System (SAS) 9.20 (SAS Institute, Cary, NC, USA) using a one-way analysis of variance (ANOVA), and multiple comparisons were performed based on the results of significance tests with the least significant difference method. Those with a *P*-value of *t*-test <0.05 and VIP ≥ 1 were considered differential metabolites between the two groups, and all the treatments had three biological replicates.

## 3 Results

### 3.1 Phosphate fertilizer affects the phenotypic and physiological characteristics of alfalfa

**Figure 1:**
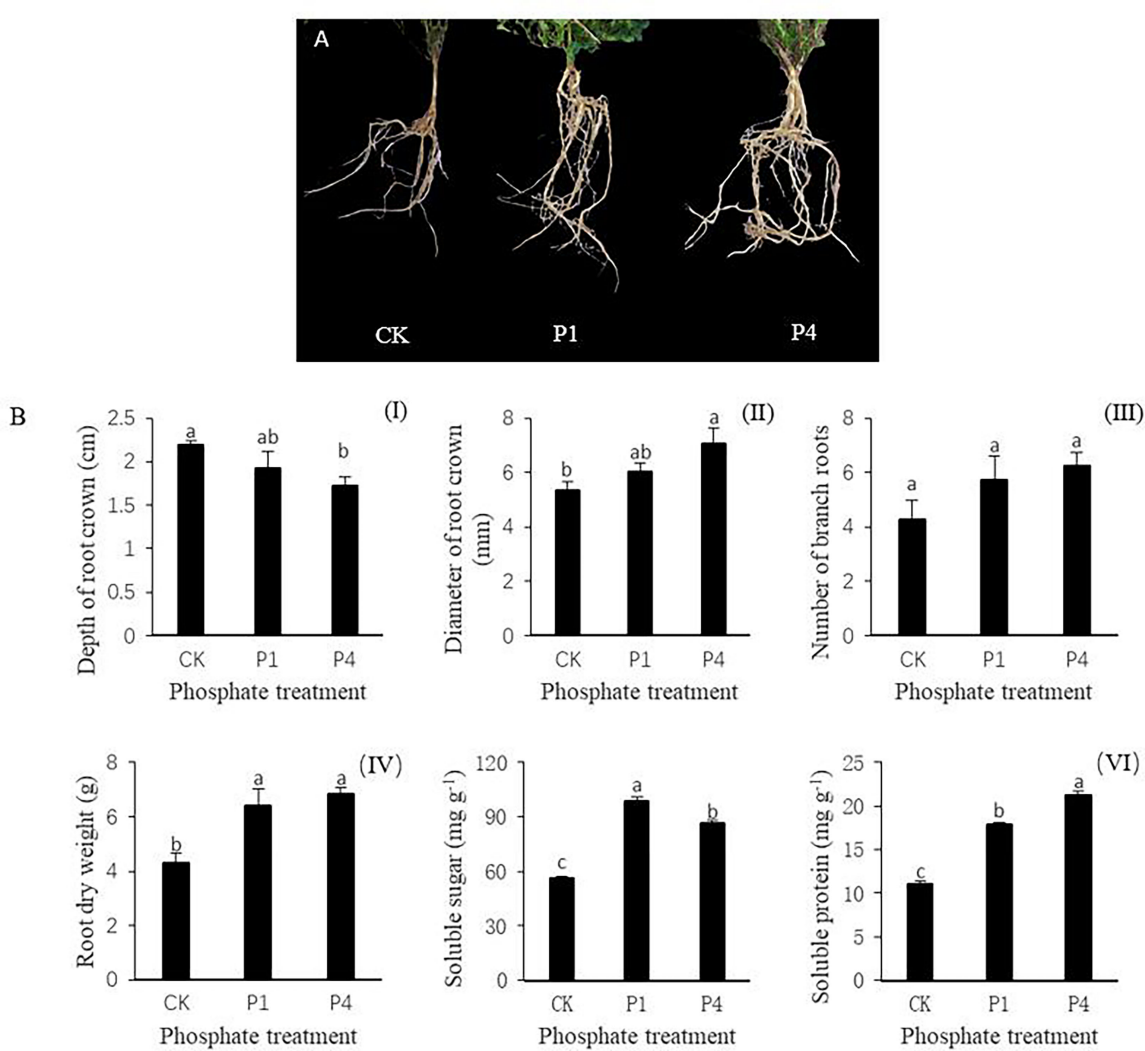
The responses of alfalfa phenotypic and physiological indices to phosphate (P) application under low temperature. **(A)** Alfalfa roots exposed to P application showed improved root growth. **(B)** Changes in the morphological and physiological indices of alfalfa roots, including (I) the depth of the root crown into the soil surface, (II) the diameter of the root crown, (III) the number of branch roots, (IV) root dry weight, (V) soluble sugar, and (IV) soluble protein. Values labeled with different lowercase letters indicate a significant difference between the P treatments at *P*< 0.05. CK, P1, and P4 represent 0, 50, and 200 P_2_O_5_ mg kg^-1^.

The phenotypes of alfalfa roots varied under different P treatments, and the growth and development of alfalfa roots were improved by the application of P (**Figure 1A**). The application of P reduced the depth of the root crown by 12.3% and 21.4% at P1 and P4 compared with the CK, respectively (**Figure 1BI**). The morphological indices of alfalfa increased during the P1 and P4 treatments compared with the CK. For example, the diameter of the root crown in the P1 and P4 treatments increased by 13.5% and 32.8%, respectively, while the number of branch roots from root crowns increased by 35.3% and 47.1%, respectively (**Figure 1BII, III**). The root dry weight increased by 48.8% and 59.3% at P1 and P4 compared with the CK, respectively (**Figure 1BIV**). The physiological indices of soluble sugar and soluble protein of the alfalfa roots increased following the application of P. The contents of soluble sugar in the P1 and P4 treatments increased by 76.6% and 54.5%, respectively, while those of the soluble protein increased by 62.5% and 92.7% compared with the CK, respectively (**Figure 1BV, VI**).

### 3.2 RNA Sequencing profiles of alfalfa roots in response to phosphate at low temperature

**Figure 2:**
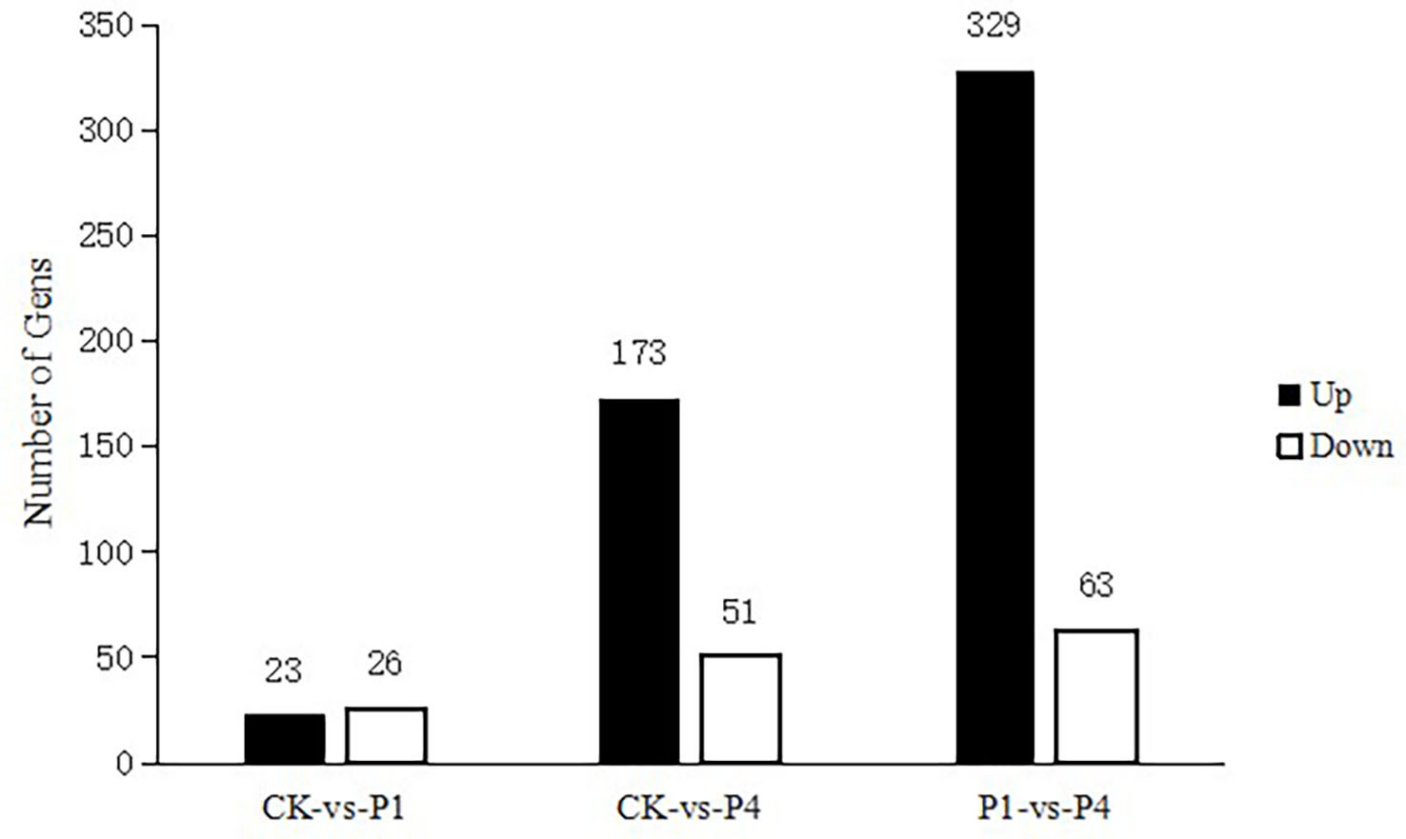
The total number of differentially expressed genes (DEGs) and upregulated and downregulated DEGs under different P treatments. DEGs, differentially expressed genes; P, phosphate.

The analysis of DEGs was performed using edge R software under different P conditions. The results are shown in **Figure 2**. Compared with the CK, 49 genes were differentially expressed under the P1 conditions, with 23 genes upregulated and 26 genes downregulated. Compared with the CK, there were 224 DEGs under the P4 conditions, with 173 genes upregulated and 51 genes downregulated. Compared with P1, there were 392 DEGs under the P4 conditions, with 329 upregulated and 63 downregulated.

**Figure 3:**
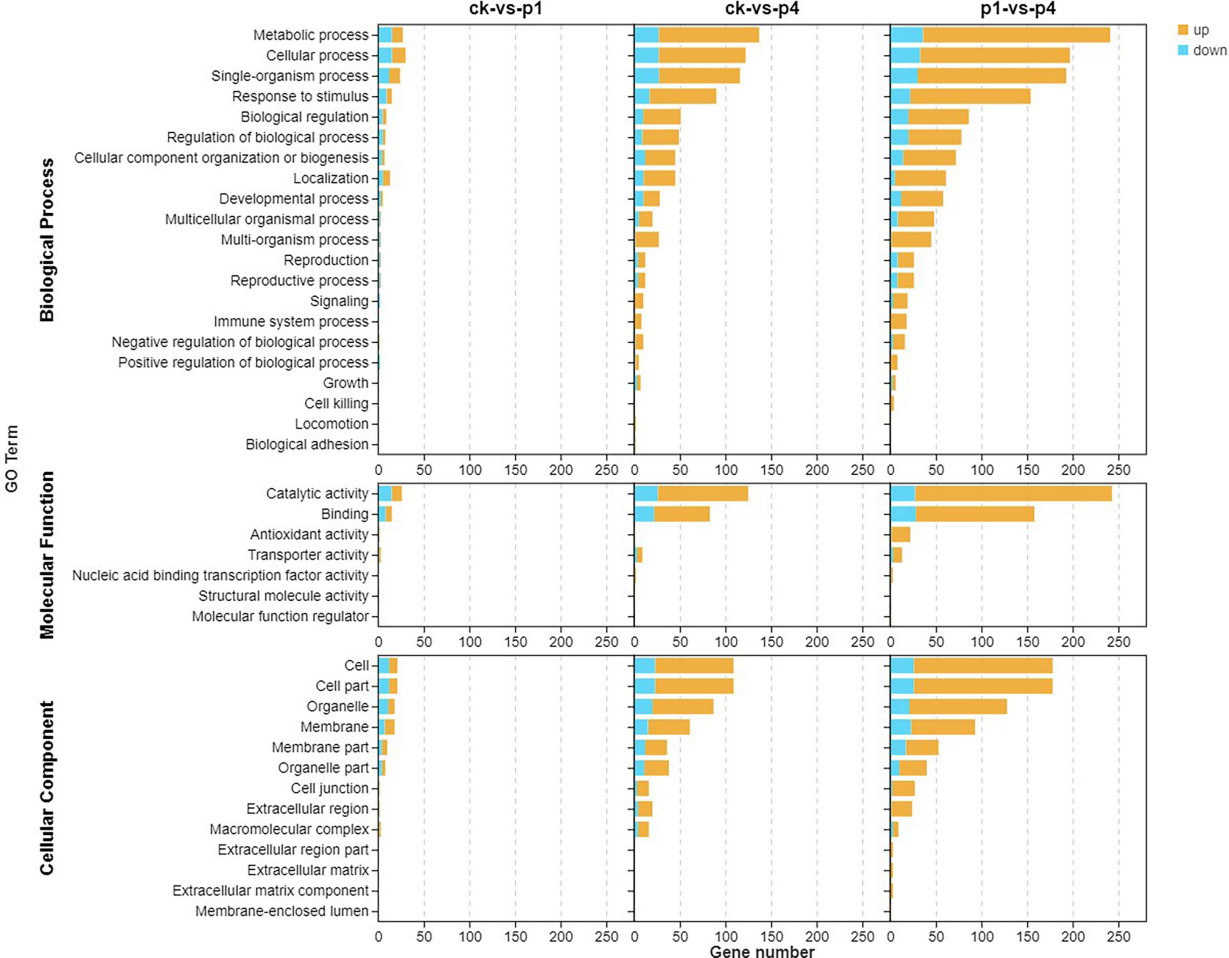
Gene Ontology (GO) enrichment analysis of the DEGs after cooling under different P treatments. DEGs, differentially expressed genes; GO, Gene Ontology; P, phosphate.

To further understand the functions and the related biological process that the DEGs participated in, GO enrichment analyses were conducted, and the results are shown in **Figure 3**. The DEGs were classified into cellular components, molecular functions, and biological processes. With the cellular component, the enriched GO terms of the three comparison groups were the cell, cell part, organelle, and membrane, membrane part, and organelle part. In addition, CK-*vs*-P4 and P1-*vs*-P4 were enriched in the cell junction, extracellular region, and macromolecular complex. Within the molecular function, the enriched GO terms of three comparison groups were catalytic activity and binding. In addition, P1-*vs*-P4 was also enriched in antioxidant activity and transporter activity. Within the biological process, the enriched GO terms of three comparison groups were metabolic process, cellular process, single-organism process, response to stimulus, biological regulation, and localization. In addition, CK-*vs*-P4 and P1-*vs*-P4 were enriched in the developmental process, multicellular organismal process, multi-organism process, reproduction, reproductive process, signaling, immune system process, and negative and positive regulation of the biological process. These results indicated that the DEGs induced by P could be enriched into multiple GO terms, and the number of GO terms and upregulated genes increased with the increase in the application of P.

**Figure 4:**
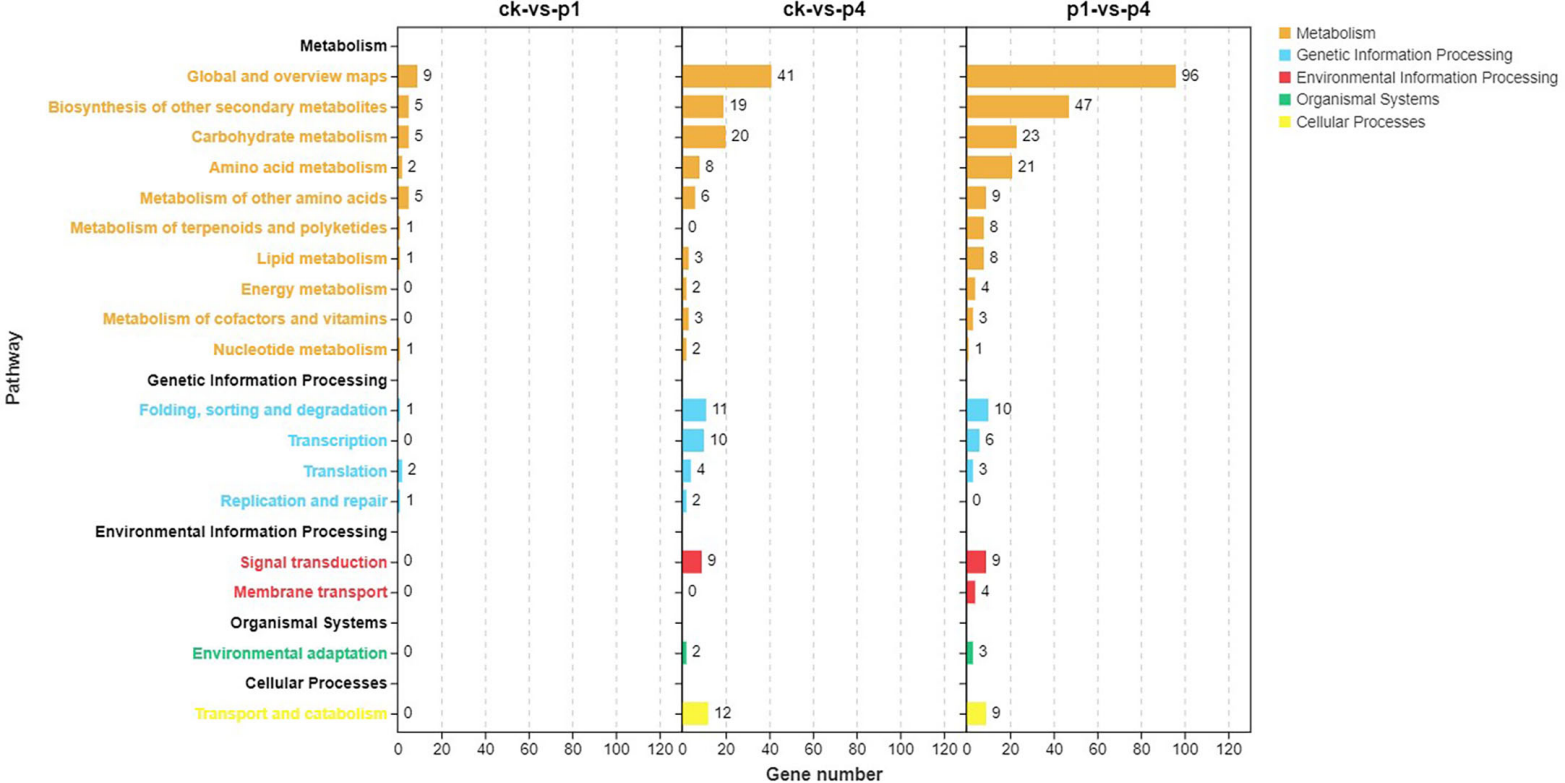
Kyoto Encyclopedia of Genes and Genomes (KEGG) pathway analysis of DEGs after cooling under different P treatments. DEGs, differentially expressed genes; KEGG, Kyoto Encyclopedia of Genes and Genomes; P, phosphate.

The pathway enrichment of the DEGs was analyzed using KEGG, and results are shown in **Figure 4**. The DEGs of CK-*vs*-P1 were primarily enriched in the biosynthesis of other secondary metabolites, carbohydrate metabolism, amino acid metabolism, and metabolism of other amino acids. The DEGs of CK-*vs*-P4 were enriched in not only the metabolic pathways described above but also the folding, sorting and degradation, transcription, and translation of genetic information processing and signal transduction and transport and catabolism. The DEGs of P1-*vs*-P4 were enriched in not only the same pathways of CK-*vs*-P4 but also the metabolism of terpenoids and polyketides and lipid metabolism. These results indicated that the number of KEGG pathways increased with the increase in P application.

### 3.3 Verification by real-time quantitative reverse transcription PCR

In addition to detecting metabolites to verify the RNA-Seq results, we used qRT-PCR to analyze six key upregulated genes to verify the results of the detection of transcriptional gene expression. The results are shown in **Figure S2**. The qRT-PCR verification results of the selected genes obtained using qRT-PCR were consistent with the expression data obtained by the corresponding RNA-seq, indicating that the RNA-seq expression data obtained are accurate and reliable.

### 3.4 Metabolite profiles of alfalfa roots in response to phosphate at low temperature

**Figure 5:**
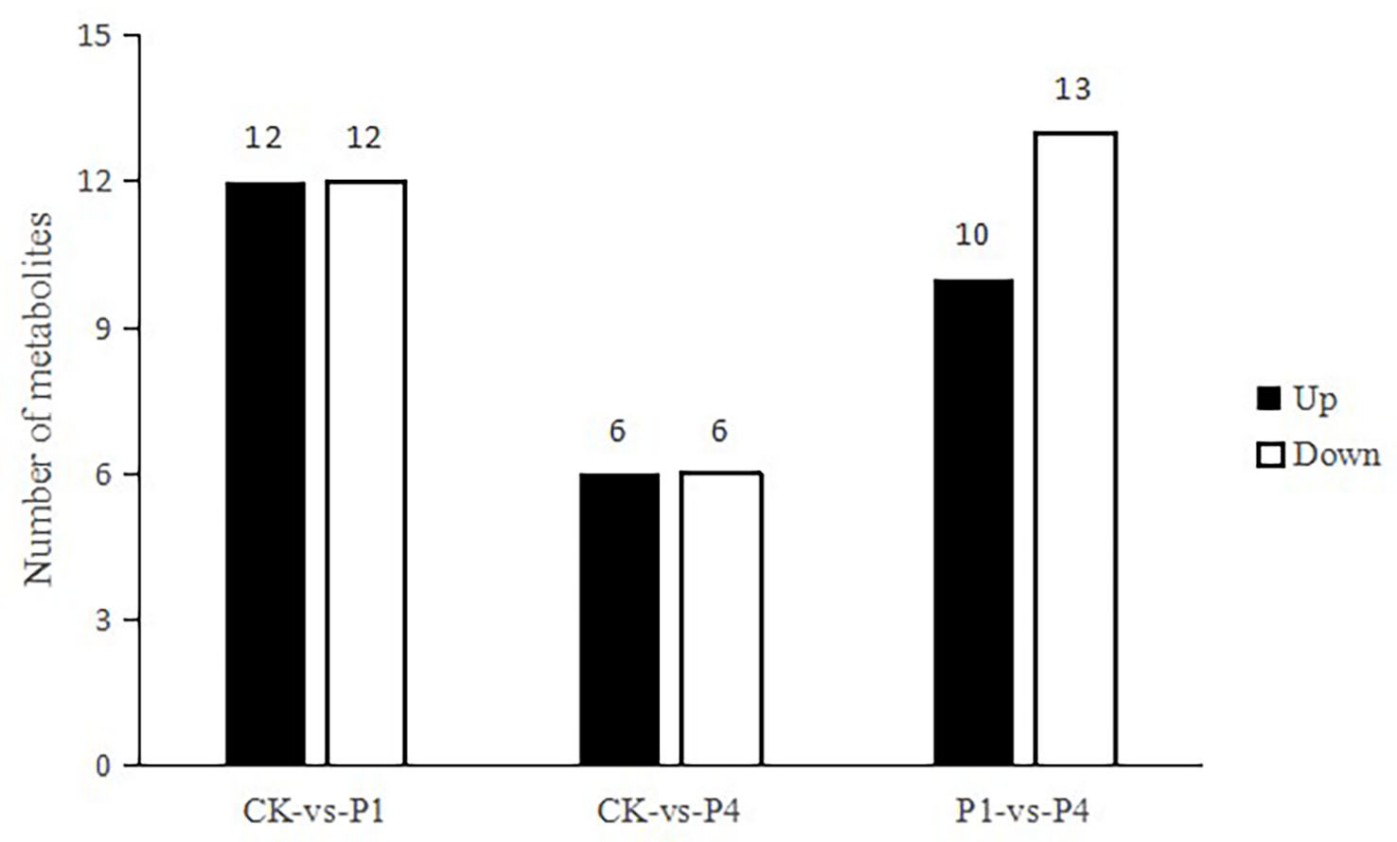
The total number of differential metabolites with differential levels, upregulated and downregulated, under different P treatments.

To obtain an overview of metabolic changes in response to the availability of P, a non-targeted metabolic analysis was performed. As shown in **Figure 5**, 24 metabolites were determined as having differential levels under P1 treatment compared with the CK. Among them, 12 metabolites increased, and 12 metabolites decreased. Compared with the CK, 12 metabolites were differentially produced under P4 treatment, and six of these metabolites increased, while six metabolites decreased. Compared with P1, 23 metabolites were determined as having differential levels under the P4 treatment. Among them, 10 metabolites increased, and 13 metabolites decreased. The primary types of metabolites that changed after the application of P were as follows: amino acids and derivatives, flavonoids, lipids, nucleotides and derivatives, organic acids, sugar, phenolic acids, and terpenoids. The types of variation in the specific metabolites and the main profile of metabolites that changed under the different P treatments are shown in **Table S2**.

**Figure 6:**
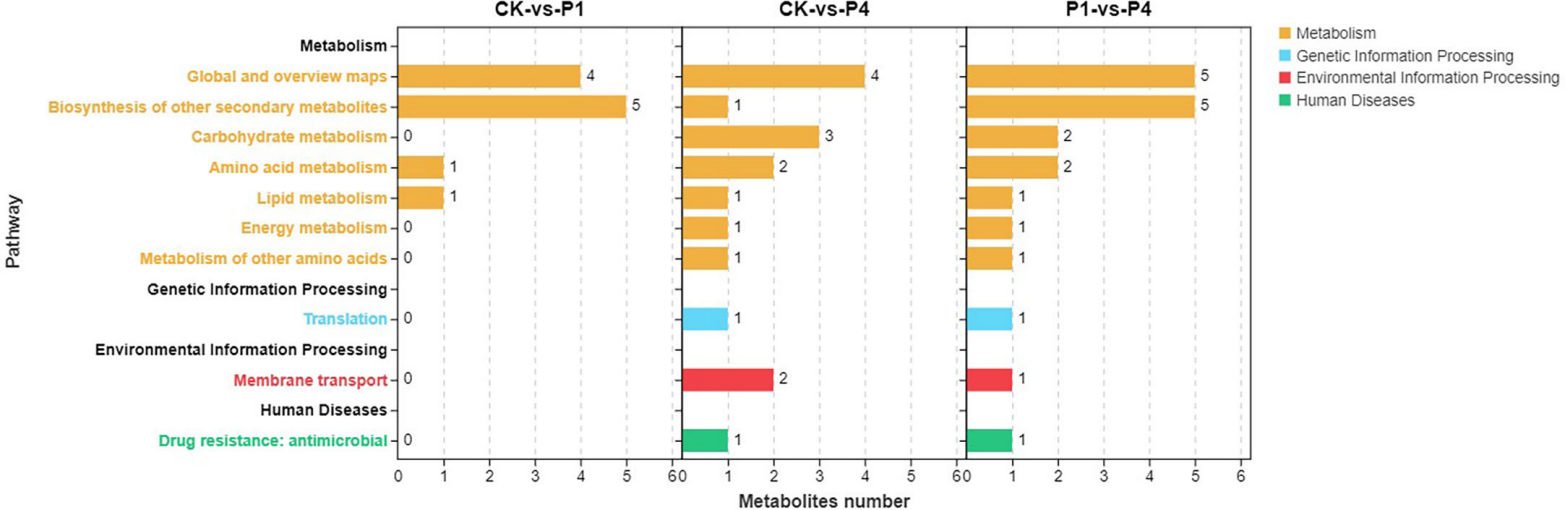
KEGG pathway enrichment analysis of the changed metabolites under different P treatments. KEGG, Kyoto Encyclopedia of Genes and Genomes.

KEGG metabolic pathway enrichment analysis classified the differential metabolites identified under low and high P treatments into metabolism, genetic information processing, environmental information processing, and human diseases (**Figure 6**). Within the metabolism, all three comparison groups were enriched in the biosynthesis of other secondary metabolites, amino acid metabolism, and lipid metabolism. In addition to these pathways, CK-*vs*-P4 and P1-*vs*-P4 were enriched in carbohydrate metabolism, energy metabolism, and the metabolism of other amino acids. These results indicated that the metabolic progress of alfalfa was significantly affected by P fertilizer at low temperature, and the application of increased amounts of P had a greater effect on metabolism.

### 3.5 Integrated metabolome and transcriptome analyses of alfalfa

**Figure 7:**
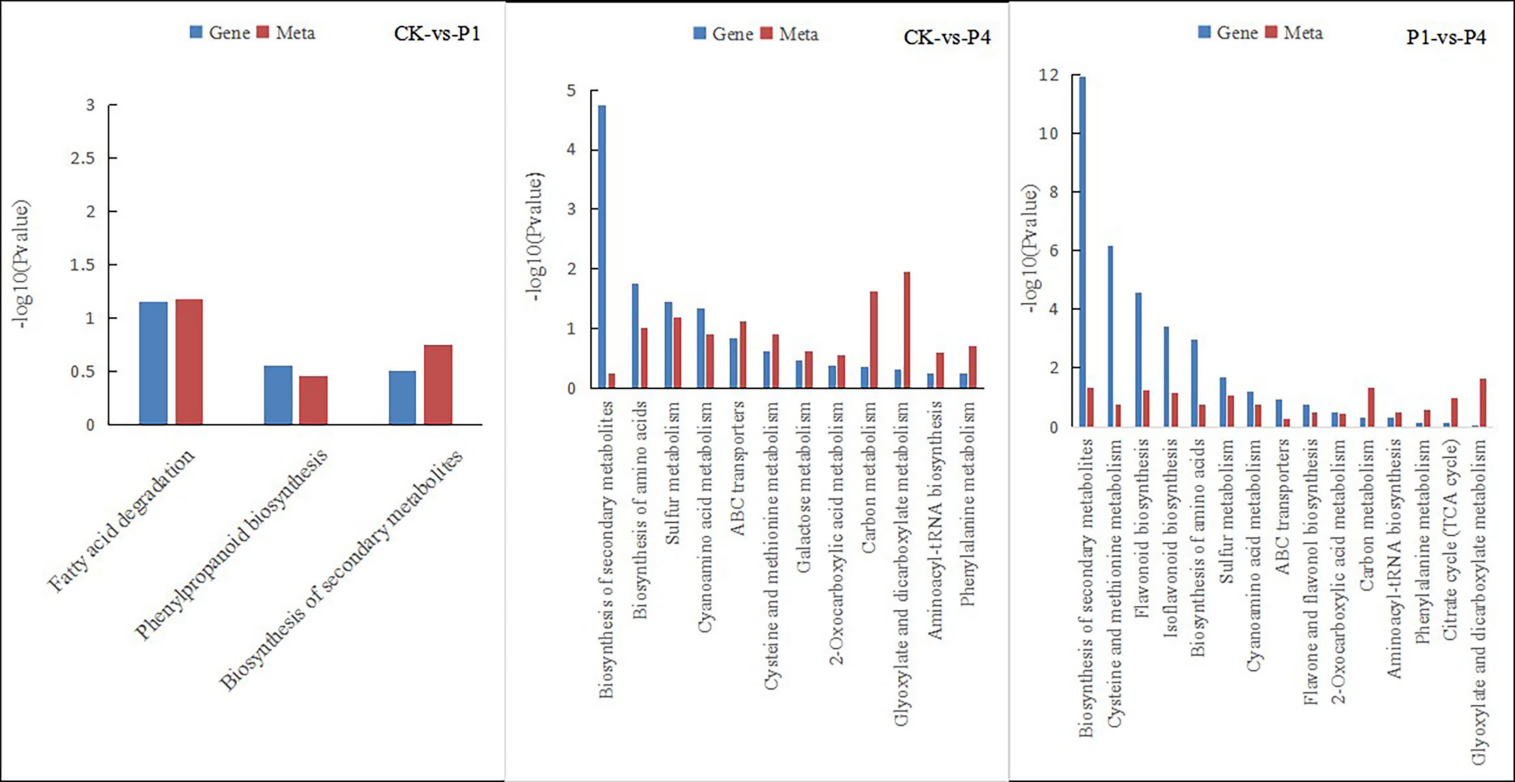
Pathways of the simultaneous annotation of differential metabolites and DEGs for different P treatments. DEGs, differentially expressed genes; P, phosphate.

The integration of the DEGs and metabolites obtained from different P levels of alfalfa identified several metabolic pathways of coenrichment (**Figure 7**). The DEGs and metabolites of the CK and P1 had three common annotated metabolic pathways, including fatty acid degradation, phenyl propionic acid biosynthesis, and the biosynthesis of secondary metabolites. The DEGs and metabolites of CK and P4 had 12 common metabolic pathways, including the biosynthesis of secondary metabolites, biosynthesis of amino acids, sulfur metabolism, cyanoamino acid metabolism, ATP-binding cassette (ABC) transporters, cysteine and methionine metabolism, galactose metabolism, 2-oxocarboxylic acid metabolism, carbon metabolism, glyoxylate and dicarboxylate metabolism, aminoacyl-tRNA biosynthesis, and phenylalanine metabolism. The DEGs and metabolites of P1 and P4 had 15 common annotated metabolic pathways, including the biosynthesis of secondary metabolites, cysteine and methionine metabolism, biosynthesis of flavonoid, biosynthesis of isoflavone, biosynthesis of amino acid, sulfur metabolism, cyanoamino acid metabolism, ABC transporter, flavonoid and flavonol biosynthesis, 2-oxocarboxylic acid metabolism, aminoacyl-tRNA biosynthesis, phenylalanine metabolism, citrate cycle, and glyoxylate and dicarboxylate metabolism. The metabolic pathways of phenyl propionic acid biosynthesis and the biosynthesis of secondary metabolites were shared by the three groups.

**Figure 8:**
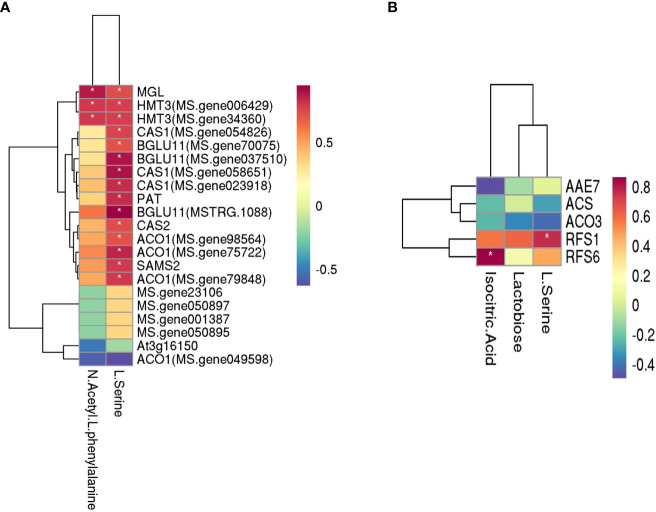
Heatmaps of amino acid metabolism and carbohydrate metabolism with the genes of three P comparison group units. **(A)** Amino acid metabolism and **(B)** carbohydrate metabolism. The darker the color (red or blue), the stronger the correlation. * means that the correlation between metabolite and gene was significant.

The related genes and metabolites in carbohydrate metabolism, amino acid metabolism, and the carbon metabolism pathway were combined in the three comparison groups with different rates of P applied, and the correlation between metabolites and genes was analyzed (**Figure 8**). Among them, N-acetyl-L-phenylalanine and L-serine are the primary metabolites that are related to amino acid metabolism, and there are 21 related genes. The level of production of N-acetyl-L-phenylalanine positively correlated with the following genes: *methionine gamma-lyase* (*MGL*) and *homocysteine S-methyltransferase 3* (*HMT3*, MS. gene006429 and MS. gene34360). The production of L-serine positively correlated with *methionine gamma-lyase* (*MGL*), *homocysteine S-methyltransferase 3*(*HMT3*, MS. gene006429 and MS. gene34360), *beta-cyanoalanine synthase* (*CAS1*, MS. gene054826, MS. gene058651 and MS. gene023918), *1-aminocyclopropane-1-carboxylate oxidase 1* (*ACO1*, MS. gene98564, MS. gene75722, and MS. gene79848), *beta glucosidase 11* (*BGLU11*, MS. gene70075, MS. gene037510, and MSTRG.1088), *protein PAT1 homolog* (*PAT*), *cysteine synthase/L-3-cyanoalanine synthase* (*CAS2*), and *S-adenosylmethionine synthase* (*SAMS2*). The primary metabolites related to carbon metabolism were lactose, isocitrate, and L-serine, and there were five related genes in which the production of lactose positively correlated with the gene *RFS6* (r = 0.62). The level of production of isocitrate did not significantly correlate with the genes, and the production of L-serine positively correlated with *RFS1* (r = 0.76).

## 4 Discussion

### 4.1 Effect of P fertilizer on the phenotypical and physiological indices of alfalfa during the increase in cold weather

Roots are the source of all the mineral elements required for plant growth; thus, root growth and development are highly plastic and vary substantially depending upon numerous soil factors (Vance et al., 2003). The root biomass is related to the accumulation of organic matter, which can contribute to improve the cold resistance of plants (Li et al., 2021). The root crown is the transitional plant structure located between the shoots and the root system, and it can influence photosynthate translocation, water transport, winter hardiness, and spring regrowth (Marquez-Ortiz et al., 1999). Alfalfa plants with deep and large root crowns survived the winter better than the plants with shallow and small root crowns (Marquez-Ortiz et al., 1999; Liu et al., 2015b; Wang et al., 2023). In addition, alfalfa with more lateral roots have strong cold resistance, and the possession of more lateral roots facilitates the absorption of more nutrients and water from the soil to meet the nutritional needs of overwintering alfalfa plants (Liu et al., 2015b). Previous research had proven the effect of a localized supply of soil P on the root proliferation of plants (Vance et al., 2003; Gruber et al., 2013). In this study, the cutting seedings had no obvious taproot, and the branch roots grew directly from the lower end of the root crown. The application of P fertilizer promoted the development of the root crown, widened the root crown, and increased the dry root weight. However, the increase in the number of branch roots was not apparent, and the depth of root crown was reduced. Our results showed that the application of P fertilizer can promote the development of the root system, particularly the root crown to some extent, and improve the cold resistance of alfalfa, which also facilitates the growth and development of alfalfa.

Soluble sugars and soluble proteins are intracellular osmotic regulators, which can be increased by stress, thus, improving the adaptability of plants to respond to stress (Zhao et al., 2015). Under low-temperature stress, amino acids, such as proline, arginine, and methionine, and soluble sugars, such as sucrose, glucose, and raffinose, accumulate in the root crowns, roots, and leaves of alfalfa (Zhang et al., 2015). In addition to reducing water loss, the enormous accumulation of these solutes can also prevent the cells from freezing and help to stabilize membrane integrity (Duca and Maria, 2015). As the amount of low-temperature stress increased, the contents of soluble protein and soluble sugar in the alfalfa root crown increased (Tao et al., 2009; Zhu et al., 2021) and improved the cold resistance of alfalfa. When the temperature increased, the resistance disappeared, and the sugar and protein content decreased (Zhang, 2008). P is an essential element in the composition of cells, which can participate in the composition of nuclear protein, lecithin, enzymes, ATP, and ADP among others and has an important impact on the synthesis, transport, and storage of sugar (Chen et al., 2013). P can enhance N fixation by legumes, promote fat metabolism, and enhance the stress resistance of crops. These changes occur because P can increase the content of soluble sugar and phospholipids in plants. Soluble sugar can reduce the freezing point of cell cytoplasm, and phospholipid can improve the adaptability of cells to temperature changes, thus, enhancing the cold resistance of crops (Wang et al., 2016). We found that the contents of soluble sugar and soluble protein both increased during exposure to increasing amounts of cold when P was applied, which is consistent with the findings of Shen et al. (2017).

### 4.2 Effect of P on the transcriptome of alfalfa roots under low-temperature stress

In general, when plants are subjected to low-temperature stress, they will change at the transcriptome level to resist the damage caused by low temperatures (Qi, 2017). Plant-specific upregulated DEGs under low-temperature stress play a more critical role in determining plant cold resistance than downregulated DEGs, and a higher percentage of upregulated genes increases the resistance of plants to cold (Zhang, 2015). In this study, we found that the P fertilizer had a substantial effect on the expression of alfalfa genes at low temperatures, and the number of DEGs and upregulated DEGs increased with the increase in P application.

Several key genes were detected in alfalfa following the application of P (**Table S1**). Cold regulated (*COR*) genes are genes that respond to low temperatures and are expressed by the regulation of a specific signal transduction pathway, and the products of *COR* genes are cryoprotective proteins that act by reducing membrane permeability during freezing and increasing the ability of membranes to expand during thawing (Wu et al., 2012). Thus, the constitutive expression of the *COR* genes could improve the freezing tolerance of plants. The novel cold-regulated genes *CsCOR1* and *CbCOR15* enhance dehydration tolerance in tobacco (*Nicotiana benthamiana*) (Li et al., 2010; Wu et al., 2012). Sangwan et al. (2001) proved that the rigidification of the plasma membrane could induce *COR* genes and result in cold acclimation. In this study, the expression of *COR* was upregulated under the application of P, which enhanced the cold resistance of alfalfa. *CYP71D10* (*P450*) is a protein-coding gene present in plants, which have an immense variety of P450s that act on different substrates. The differential activities of P450s are believed to represent one of the mechanisms that enables certain crops species to be more tolerant of abiotic stress than other crops, particularly following herbicide treatment (Siminszky et al., 1999). Our research proved that the application of P under low temperatures could enrich the expression of a *P450* that plays a vital role in cold tolerance. *PAT* is a photonuclear aspartate aminotransferase, which has not only the activity of ASPAT but also the activity of prephenate transaminase. It is primarily located in the cytoplasm, mitochondria, and plastids and is involved in not only plant metabolism but also the abiotic stress response pathways of plants (Wei et al., 2022). *PAT* reduces the accumulation of ROS and protects the structural integrity of cell membranes, which improves the abiotic tolerance stress of plants (Tian, 2021). *S-adenosyl methionine synthetase* (*SAMS*) uses L-methionine as a substrate, and ATP provides the energy to catalyze the synthesis of S-adenosylmethionine, which plays a very important role in the responses of plants to stress (Roje, 2006). Zhang et al. (2020) confirmed that the overexpression of *SlSAMS1* significantly reduced the accumulation of superoxide ( ${\text{O}}_{2}^{\cdot -}$ ), hydrogen peroxide (H_2_O_2_) and malondialdehyde and enhanced the contents of abscisic acid and the enzymes that scavenge ROS, including superoxide dismutase, catalase, and ascorbate peroxidase, and plays an important role in improving the drought and salt tolerance of transgenic tomato (*Solanum lycopersicum* L.). Guo et al. (2014) showed that the overexpression of *MfSAMS1* promoted polyamine synthesis and oxidation, which, in turn, improved the induction of protection against antioxidants by H_2_O_2_. As a result, this enhanced the tolerance to freezing and chilling stress in transgenic alfalfa plants. Our study proved that the expression of *SAMS* in the root crown of alfalfa treated with P was enhanced, which improved the plant’s resistance to cold. *HSP70* plays an important role in plant growth and development and responds to various abiotic stresses, such as heat, drought, salinization, hormones, and other environmental perturbations (Cho and Choi, 2009; Montero-Barrientos et al., 2010; Usman et al., 2017). In heat-tolerant varieties of pepper (*Capsicum annuum* L.), the level of expression of *CaHsp70* was rapidly upregulated upon heat stress, and the thermal stability of biofilm was simultaneously enhanced (Usman et al., 2015). Lv et al. (2022) found that the expression of *JcHsp70s* and its corresponding miRNAs, which are highly responsive to low temperature, is generally negatively regulated, and the interaction between *JcHsp70s* and miRNAs may be involved in the process of improving the cold tolerance of purging nut (*Jatropha curcas* L.) under low temperatures. In this study, treatment with P fertilizer also increased the expression of *HSP70* in the root crown of alfalfa under low-temperature stress. In addition, there are other DEGs owing to the application of P fertilizer. These DEGs were enriched in three metabolic pathways, including the biosynthesis of other secondary metabolites, carbohydrate metabolism, and amino acid metabolism. They can also be involved in scavenging excess ROS, supplying energy, and maintaining cell osmotic pressure under low-temperature stress, which are very important for alfalfa to survive over the winter.

### 4.3 Effects of phosphate on the metabolism of alfalfa roots under low-temperature stress

The changes in plant metabolites under stress can reflect the ability of plants to adapt to stress. Plants accumulate different sugars and amino acids under low-temperature stress, such as trehalose, glucose, fructose, inositol, galactitol, raffinose, sucrose, putrescine, ascorbate, phenylalanine, and alanine (Jin et al., 2017). Under the influence of external substances, the expression of metabolites in plants will also change after responding to low-temperature stress (Zhou et al., 2017). Flavonoids are important secondary metabolites, which can enhance plant resistance and chemical defenses, thus, improving the tolerance of plants to abiotic stress (Zhu et al., 2007; Liu et al., 2015a). When plants are injured, secondary metabolites, such as alkaloids and polyphenolic acids, will gradually accumulate and form defense structures, thus, enhancing plant resistance (Hu et al., 2012). Terpenoids can directly or indirectly participate in a series of biological processes, such as hormone synthesis, cell membrane stability, and photosynthesis in plants. Plants can improve their ability to protect and defend themselves by regulating the content of terpenoids. Sugar metabolism is a critical metabolic cycle process in plant growth and development, and providing energy is the primary function of sugar metabolism. Secondly, sugar synthesis metabolism can also play an important role in the response of plants to abiotic stress (Sambe et al., 2015). Plants with low external levels of Pi have serious problems maintaining a balanced ratio of sugars, lipids, amino acids, organic acids, terpenes, and flavonoids, but the application of P can improve this situation (Mo et al., 2019; Ding et al., 2021; Nasr Esfahani et al., 2021). In this experiment, the application of P could regulate the types and contents of metabolites. When a small amount of P fertilizer is applied, it primarily affects the biosynthesis of secondary metabolites, while the application of more P fertilizer primarily affects the biosynthesis of secondary metabolites, amino acid metabolism, carbohydrate metabolism, and others.

### 4.4 Integrated metabolome and transcriptome analysis revealed the mechanism used by phospate to improve cold tolerance

The mechanism of cold resistance of alfalfa is very complex and involves morphology, physiology and biochemistry, molecular, transcription, and metabolism. Hence, it is crucial to integrate a variety of combinatorial techniques to comprehensively analyze the mechanism of the cold resistance of alfalfa (Wang and Xu, 2021). During cold acclimation, root morphology and the synthesis of compatible solutes were closely related to the cold resistance of alfalfa. Less root mass and lower concentrations of root total non-structural carbohydrates can minimize the survival of plants over the winter when the plants are subjected to low temperatures (Castonguay et al., 2006). The crown depth below the soil surface is viewed as a key morphological adaptation for forage legumes to overwinter successfully. The alfalfa varieties that have more branch rooting in general were better able to resist cold than the varieties that have a taproot or fewer types of branch roots (Johnson et al., 1998). The accumulation of soluble sugars under low temperature plays an important role in the acquisition of cold tolerance in plants (Guy, 1990). Several proteins were increasingly or newly synthesized during the acclimation of alfalfa to the cold (Mohapatra et al., 1987). Simultaneously, free amino acids were shown to accumulate in the taproots and crowns (Hendershot and Volenec, 1993; Dhont et al., 2003). The increase in free proline was notable, and the concentrations of arginine and histidine were also markedly increased (Dhont et al., 2003). In this study, the application of P fertilizer significantly changed the traits of root system architecture and increased the contents of soluble sugar and protein during the cold accumulation period. In addition, there were commonly annotated pathways shared by the transcriptome and metabolome. Thus, the application of P fertilizer influences the cold tolerance of alfalfa. With the increase in the application of P, the common metabolic pathways for the enrichment of DEGs and metabolites affected by P gradually increased. The biosynthesis of amino acids is regulated by a compound metabolic network that links N assimilation with carbon metabolism (Hans et al., 2008). Carbohydrate metabolism is the most important basic metabolism in the plant, which provides the necessary carbon frame and energy to synthesize amino acids, proteins, and nucleic acids in N metabolism. Under low-temperature stress, the application of P primarily affected the contents of L-serine, N-acetyl-L-phenylalanine, isocitrate, and lactobiose. A correlation analysis revealed that the production of L-serine positively correlated with *MGL*, *HMT3*, *CAS1*, *BGLU11*, *PAT*, *CAS2*, *ACO1*, *SAMS2*, and *RFS1*. N-acetyl-L-phenylalanine positively correlated with *MGL* and *HMT3*, and lactobiose positively correlated with *RFS6*. Among them, *RFS1* and *RFS6* are related to the synthesis of raffinose synthase, which is a crucial enzyme in the raffinose metabolism pathway. This pathway can catalyze the reaction of inositol galactoside and sucrose to raffinose, and they can also catalyze the reaction of inositol galactoside and raffinose to synthesize stachyose. Its expression can be regulated by various stress response signals and transcription factors (Nishizawa et al., 2008; Cho et al., 2010). *SAMS2* facilitates the synthesis of S-adenosyl methionine synthetase and can be induced by a variety of stress factors; its overexpression can significantly improve the resistance to plant stress (Guo et al., 2014). The overexpression of *PAT* increased the contents of proline and soluble sugar in transgenic *Arabidopsis thaliana* and improved the cold, drought, and saline–alkali tolerance of plants (Yuan et al., 2016). Other genes also play essential roles in the synthesis and metabolism of amino acids and carbohydrates. Thus, the moderate application of P fertilizer can improve the cold resistance of alfalfa by regulating the synthesis and metabolism of amino acids and sugars.

## 5 Conclusion

Low-temperature stress affects the growth and development of alfalfa, leading to morphological, physiological, metabolic, and molecular changes and, thus, limits the overwintering and production of alfalfa. The application of P can improve the cold resistance and forage yield of alfalfa. In this study, we analyzed the changes of alfalfa under low-temperature stress by transcriptomic and metabolomic approaches after the application of P. We found that the application of P fertilizer improved the root structure and increased the content of soluble sugar and soluble protein in the root crown, and there were 49 DEGs (23 genes upregulated) and 24 metabolites (12 upregulated) following 50 mg kg^-1^ of P applied and 224 DEGs (173 genes upregulated) and 12 metabolites (6 upregulated) following 200 mg kg^-1^ of P applied, respectively, compared with the CK. These genes and metabolites were significantly enriched in the biosynthesis of other secondary metabolites, carbohydrate metabolism, and amino acid metabolism pathways. The integration of the transcriptomic and metabolomic analyses showed that P could affect the biosynthesis of N-acetyl-L-phenylalanine, L-serine, lactose, and isocitrate and the expression of related genes to regulate the cold tolerance of alfalfa. Our findings could contribute to a deeper understanding of the mechanism that alfalfa uses to respond to cold tolerance and lay a theoretical foundation for breeding highly P-efficient alfalfa.

## Data availability statement

The data presented in the study are deposited in the NCBI repository, accession number PRJNA909902. The data has been released.

## Author contributions

YW, LY and JX designed the experiment. YW, ZS and QW collected the samples in the field, and YW and ZS conducted laboratory analyses. YW analyzed the data, interpreted the results, and wrote the manuscript. All authors reviewed and edited the manuscript and approved the submitted version.

## Footnotes

1
http://www.r-project.org/

2
http://www.massbank.jp/

3
http://www.hmdb.ca/

4
http://www.ab.wur.nl/moto/

5
http://metlin.scripps.edu/index.php
