# Supplementary material for: Transcriptomics and metabolomics revealed that phosphate improves the cold tolerance of alfalfa
Source: Front Plant Sci. 2023 Mar 9;14:1100601. doi: 10.3389/fpls.2023.1100601 (PMC10034057; doi:10.3389/fpls.2023.1100601)
Supplement: Supplementary file 1 [file DataSheet_1.docx]

Supplementary Material

# Supplementary Figures and Tables

## Supplementary Figures


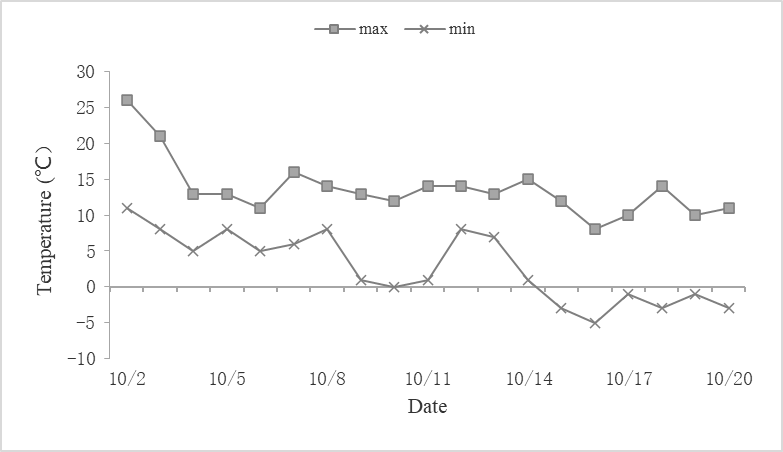


**Supplementary Figure 1** Maximum (max.) and minimum (min.) temperatures during the sampling period


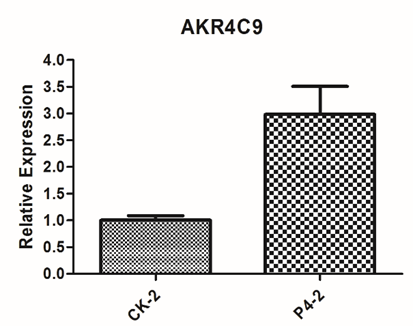

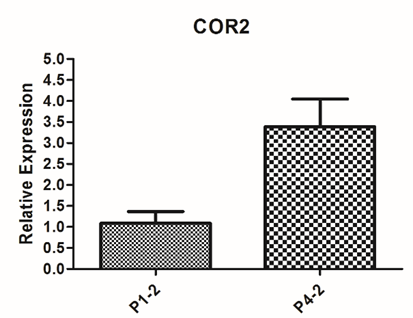


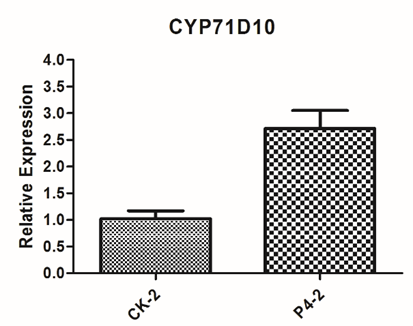

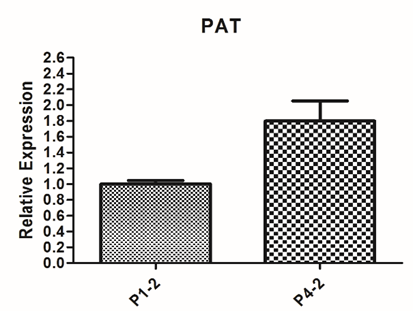


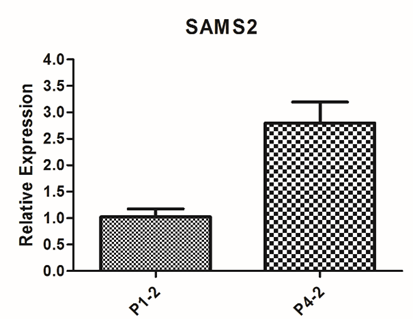

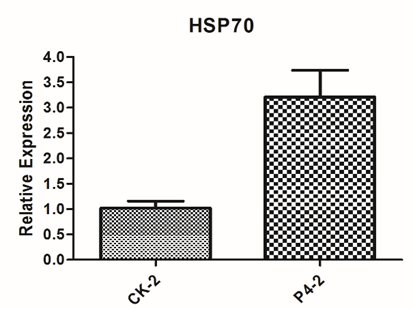


**Supplementary Figure 2** The qRT-PCR validation of differential gene expression levels

## Supplementary tables

**Supplementary Table 1** The genes and primers used for qRT-PCR analysis

| Gene | Forward primers | Reverse primers |
| --- | --- | --- |
| AKR4C9 | GGACACAGCGTGCTACCTAAGA | CACCATCCCAGAGTTCTTCAAT |
| COR2 | AGTAGTCCTCCCCTCCTCCA | GCTTCTCCAACAGATTTTTCCA |
| CYP71D10 | ACAAAACAAAGCACGCAAGGA | CGGACGATGTTTCGCTACCA |
| PAT | CCCCCGCAACGCATACAA | GGTAGAAACAGCCTCCCCAC |
| SAMS2 | ATGGCAGCAGAGACCTTCC | TCAGCATCAAGACCTACATCATC |
| HSP70 | GGCTGGAAAAGGTGAAGGAC | CAGGCACAGTTACGACAGC |
| AC101 | GTTCCGAGAGATTCCGTTGC | ATGCCAAGATAGACCCACCA |

**Supplementary Table 2** The major profile of expression of changed metabolites in different P treatments

| Class | Compounds | CK-vs-P1 | CK-vs-P4 |
| --- | --- | --- | --- |
| Amino acids and derivatives | L-Serine | 1.11 | 2.49 |
|  | N-Acetyl-L-phenylalanine | 0.67 | 1.10 |
| Flavonoids | 3,9-Dihydroxypterocarpan | 1.32 | 1.75 |
|  | Glycitein | 2.01 | 0.58 |
|  | Kaempferol | 2.28 | 0.96 |
|  | Eriodictyol | 13.21 | 0.24 |
|  | Afrormosin | 1.48 | 0.60 |
|  | 4'-Hydroxy-5,7-dimethoxyflavanone | 1.15 | 1.29 |
|  | Tricetin | 4.48 | 0.92 |
|  | 4,4'-dihydroxy-2,6-dimethoxydihydrochalcone | 3.43 | 0.89 |
|  | 5,7,4'-Trimethoxyflavone | 11.61 | 10.17 |
|  | 5,7-Dihydroxy-2'-methoxy-3',4'-methyleneoxydihydroisoflavone | 4.61 | 0.88 |
|  | 3,3',5-Trihydroxy-4',7-dimethoxyflavanone | 7.40 | 1.35 |
|  | Formononetin-7-O-glucoside (Ononin) | 1.30 | 0.80 |
|  | Chrysoeriol-7-O-(6''-malonyl)glucoside | 1.46 | 0.41 |
|  | Apigenin-6,8-di-C-glucoside (Vicenin-2) | 1.83 | 0.69 |
| Lipids | Palmitaldehyde | 1.12 | 1.17 |
|  | LPE 16:0/18:2 | 0.71 | 1.15 |
|  | LPC 16:0/18:3/18:2 | 0.80 | 1.25 |
| Nucleotides and derivatives | Ribosyladenosine | 0.55 | 1.09 |
| Organic acids | TranexamicAcid | 1.77 | 1.02 |
|  | Isocitric Acid | 0.99 | 1.27 |
| Sugar | Lactobiose | 0.82 | 1.42 |
| Phenolic acids | 4-Nitrophenol | 1.23 | 1.25 |
|  | 2,5-Dihydroxyacetophenone | 2.17 | 0.64 |
|  | Coniferyl alcohol* | 2.47 | 0.99 |
|  | Syringaldehyde | 2.16 | 0.55 |
|  | Protocatechuic acid-4-O-glucoside* | 1.52 | 0.52 |
|  | 1-O-Glucosyl sinapate | 7.83 | 2.38 |
| Terpenoids | Soyasapogenol E | 0.54 | 0.87 |
|  | Ursolic acid | 0.54 | 0.87 |
|  | Soyasaponin | 0.48 | 0.43 |
|  | Dehydrosoyasaponin | 0.44 | 0.57 |
|  | Soyasapogenol | 0.44 | 0.53 |
|  | Bayogenin | 0.50 | 0.67 |
